# Death of Professor E. Deming

**Published:** 1855-03

**Authors:** 


### DEATH OF PROFESSOR E. DEMING.

We regret to learn of the death of Dr. Deming, who as a gentleman and physician, stood deservedly high wherever he was known. This event took place on the 23d of February, at his residence in Lafayette, Ind., in the 58th year of his age.

Dr. Deming has occupied for several years past the chair of Pathological Anatomy and Clinical Medicine, in the Medical Department of the University of Missouri. He is said to have filled the position with credit to himself and great satisfaction to those with whom he was associated.

Dr. Deming's name also appeared as one of the editors of our valued cotemporary, the St. Louis Medical and Surgical Journal, but, owing to his not residing in St. Louis, he never took an active part either as editor or a contributor to that Journal.

